# Can Polyphenols Inhibit Ferroptosis?

**DOI:** 10.3390/antiox11010150

**Published:** 2022-01-12

**Authors:** Marija Lesjak, Nataša Simin, Surjit K. S. Srai

**Affiliations:** 1 Department of Chemistry, Biochemistry and Environmental Protection, Faculty of Sciences, University of Novi Sad, Trg Dositeja Obradovića 3, 21000 Novi Sad, Serbia; marija.lesjak@dh.uns.ac.rs (M.L.); natasa.simin@dh.uns.ac.rs (N.S.); 2 Division of Biosciences, Research Department of Structural and Molecular Biology, University College London, Darwin Building, Gower Street, London WC1E 6BT, UK

**Keywords:** polyphenols, metabolites, ferroptosis inhibition

## Abstract

Polyphenols, a diverse group of naturally occurring molecules commonly found in higher plants, have been heavily investigated over the last two decades due to their potent biological activities—among which the most important are their antioxidant, antimicrobial, anticancer, anti-inflammatory and neuroprotective activities. A common route of polyphenol intake in humans is through the diet. Since they are subjected to excessive metabolism in vivo it has been questioned whether their much-proven in vitro bioactivity could be translated to in vivo systems. Ferroptosis is a newly introduced, iron-dependent, regulated mode of oxidative cell death, characterized by increased lipid peroxidation and the accumulation of toxic lipid peroxides, which are considered to be toxic reactive oxygen species. There is a growing body of evidence that ferroptosis is involved in the development of almost all chronic diseases. Thus, ferroptosis is considered a new therapeutic target for offsetting many diseases, and researchers are putting great expectations on this field of research and medicine. The aim of this review is to critically analyse the potential of polyphenols to modulate ferroptosis and whether they can be considered promising compounds for the alleviation of chronic conditions.

## 1. Overview on Polyphenols

**Figure 1:**
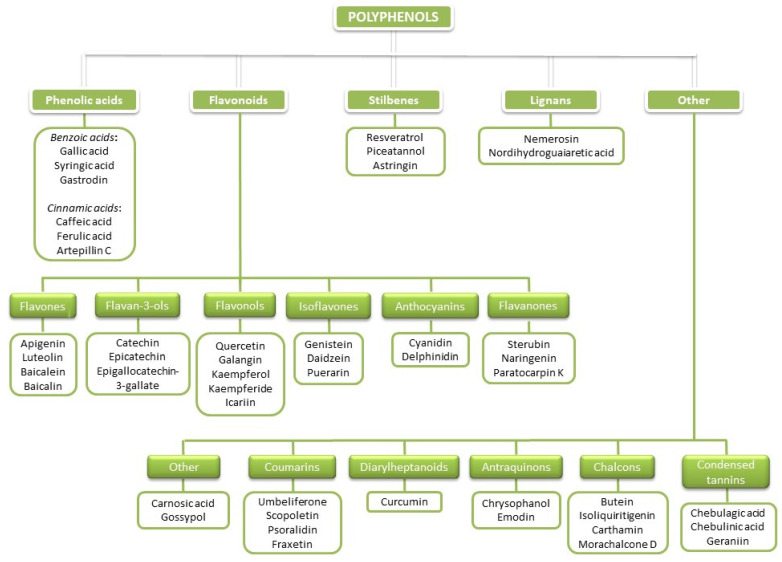
Basic classification of PCs with representatives of particular classes.

**Figure 2:**
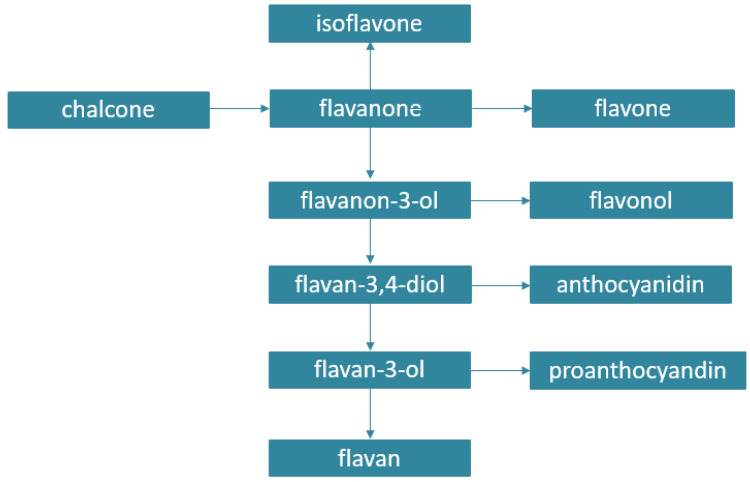
Biosynthetic origin of flavonoids.

**Figure 3:**
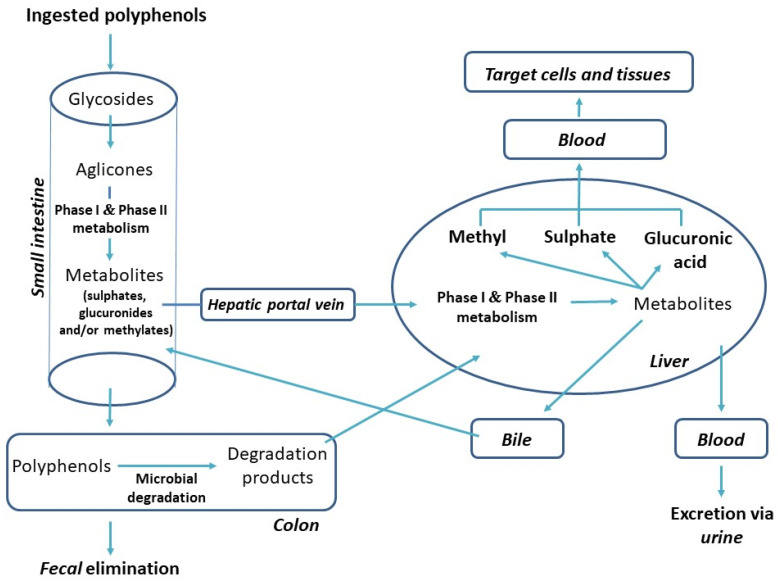
Biotransformation of PCs after oral intake [13].

**Figure 4:**
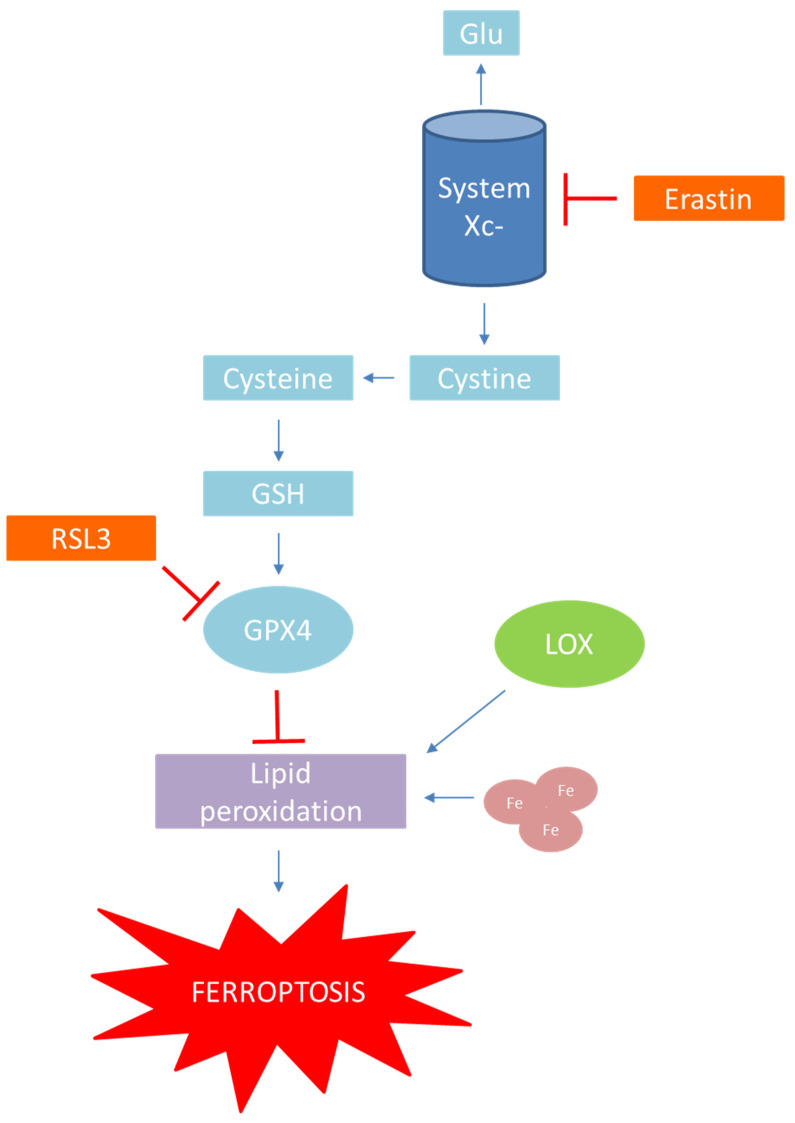
Mechanisms of ferroptosis induction and inhibition.

**Figure 5:**
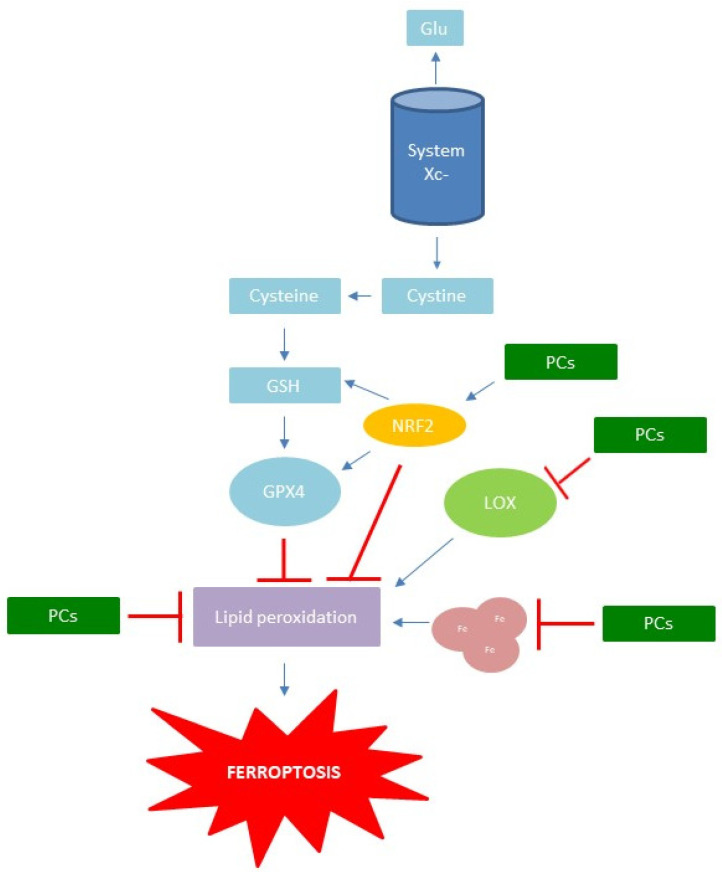
Mechanisms of ferroptosis inhibition by PCs.

Polyphenols (PCs) are a large heterogeneous group of naturally occurring molecules commonly found in higher plants. PCs have been studied extensively during the past 40 years due to their large spectrum of potential beneficial effects on human health, improving quality of life and prolonging lifespans. PCs are products of plant secondary metabolism that are synthesized to protect plants from different environmental factors and to enable their communication with the environment. More than 10,000 PCs have been identified in various plant species [1]. There is a great chemical structural diversity among PCs, but their common characteristic is the presence of more than one phenolic hydroxy group attached to one or more aromatic rings. According to the number of phenolic rings and the structural elements that link these rings, PCs are subdivided into several classes. The basic classification of PCs would include five main polyphenolic classes: phenolic acids (benzoic (C6-C1) and cinnamic acids (C6-C3)); flavonoids (C6-C3-C6); stilbenes (C6-C2-C6), lignans ((C6-C3)2); and others (coumarins (C6-C3), diarylheptanoids (C6-C7-C6), anthraquinones (C6-C2-C6), naphthoquinones (C6-C4), xanthones (C6-C1-C6), and condensed tannins (C6-C3-C6)n; Figure 1; [2,3,4,5])).

Phenolic acids and flavonoids are widely distributed in the plant kingdom, while other classes such as lignans, stilbenes, anthraquinones, coumarins, or diarylheptanoids are more specific to particular plant genera. Phenolic acids include hydroxycinnamic acids and derivatives of benzoic acid. Flavonoids represent a subclass of PCs with a C6-C3-C6 backbone structure, and they can be subdivided into two major groups according to whether the central heterocyclic ring is unsaturated or not. Flavonoids with an unsaturated central ring include anthocyanins, flavones, and flavanols, while flavanones and flavans belong to a group with saturated rings. Flavonoids in which the 2-phenyl side chain is isomerized at the 3-position are known as isoflavonoids. Plant PCs are synthetized from carbohydrates via the shikimate pathway. The biosynthetic relationship of flavonoids is shown in Figure 2. [6].

The majority of flavonoids are monomeric, while flavan-3-ols (catechins) can oligomerize—giving proanthocyanidins—or polymerize, giving non-hydrolysable tannins.

PCs mostly occur in a conjugated form as glycosides, with sugar residues linked predominantly to the hydroxy groups or directly to an aromatic carbon, or as esters with organic acids. They can also be present in plants in a free form, but much less frequently.

### Food Sources of PCs

PCs are widely present in foods of plant origin. They are found in all fruits and vegetables, but with a qualitative and quantitative distribution that varies between different plants. Since PCs are present in plants as complex mixtures, only a small number of species have been examined systematically for their phenolic composition; thus, the data are usually incomplete and there is no comprehensive database on PC amounts in food [3]. Thus, it is hard to precisely estimate the dietary intake of PCs in a particular diet or to assess the dietary intake in a population. The most abundant PCs in the diet are phenolic acids and flavonoids. According to some rough estimations, the mean daily total flavonoid intake in the U.S. is 189.7 mg/d, from which there are listed classes present in different percentages: flavan-3-ols (83.5%), followed by flavanones (7.6%), flavanols (6.8%), anthocyanidins (1.6%), flavones (0.8%), and isoflavones (0.6%) [7].

Quercetin and kaempferol are the most abundant flavanols in plant diet. Flavanols are mainly present in plants in their highly hydrophilic glycosylated forms, mainly as β-glycosides of various sugars (monosaccharides—galactose, glucose, arabinose, xylose and rhamnose, and the disaccharide rutinose) [8]. The dominant quercetin glycosides are quercetin-3-*O*-galactoside (hyperoside), quercetin-3-*O*-rhamnoside (quercitrin), quercetin-3-*O*-rutinoside (rutin), quercetin-3-*O*-glucoside (isoquercitrin), and quercetin-4′-*O*-glucoside [9].

High concentrations of quercetin are found in onions, tea, apples, asparagus, berries and red wine, while the richest plant sources of kaempferol are green leafy vegetables, including spinach and kale, and herbs such as dill, chives, and tarragon [10,11]. Quercetin can also be taken as a dietary supplement with a daily recommended doses of 200–1200 mg, as well as through functional foods with a concentration range of 10–125 mg per serving. Dietary supplementation with quercetin and its addition into food is highly supported by data on its safety [12].

Apigenin and luteolin are the main representatives of flavones, the less common class of flavonoids. Rich natural sources of these compounds are green leafy herbs, such as celery, parsley, and spinach, as well as chamomile, green paper, eggplant, oranges, and red wine [13]. In these sources, apigenin is commonly found as a 7-*O*-glucoside, 6-C-glucoside, or 8-C-glucoside. The daily intake of apigenin in the human diet is between 0.45 and 1.17 mg [14].

Naringenin and hesperidin are flavanones specific to citrus fruits. For citrus flavonoids, anti-inflammatory, anticarcinogenic, and antitumor activities have been reported. Naringin mainly occurs as glycosides such as naringenin-7-rhamnoglucoside (naringin) or naringenin-7-glucoside. Naringin is present in grapefruit and grapefruit juices in high amounts (from 100 to 500 mg/L of juice) and is responsible for the bitterness of grapefruit juices [15].

Catechin and epicatechin are most commonly found monomeric flavan-3-ols. Their main food sources are fruits (berries, cherries, apple, grapes, plums, apricots), teas, red wine, and cocoa. The average daily monomeric catechin and epicatechin intake is 9–13 mg and 11–17 mg, respectively [16,17]. Catechin and epicatechin are often found in fruits and vegetables in a polymerized form, such as oligomeric and polymeric proanthocyandins (condensed tannins).

Resveratrol is the most investigated polyphenol from the stilbene family. It is found in more than 70 plant species—often as a glucoside—particularly in grape skin and seeds, berries, apples, plums, and peanuts, as well as in food products such as red wine. The total dietary intake of resveratrol is up to 4 mg/day [18]. Resveratrol can also be taken as a dietary supplement, with daily recommended doses of 250–1000 mg for up to 3 months. Numerous beneficial health effects have been confirmed for resveratrol, including anticancer, antimicrobial, neuroprotective, antiaging, anti-inflammatory, cardioprotective, and blood-sugar lowering properties, as well as life-prolonging effects [19].

Diarylheptanoid curcumin, the major bioactive component of the rhizome of turmeric (*Curcuma longa* L.) has been extensively studied over the past few decades. The average intake of turmeric in the Indian diet is approximately 2–2.5 g, which corresponds to a daily intake of approximately 60–100 mg of curcumin [20]. It can also be taken as a dietary supplement, with daily doses of up to 12 g. Numerous biological activities, such as anti-inflammatory, antioxidant, hypoglycaemic, wound healing, antimicrobial, and antitumor activities have been confirmed for this compound [21].

For most PCs, dietary recommendations for their intake have not been established yet.

## 2. Health Benefits of PCs

Epidemiological studies have indicated that intake of polyphenol-rich food, such as fruits, vegetables, and cereals, is directly related to declines in the incidence of chronic diseases in the population, such as cardiovascular disease, obesity, diabetes mellitus, asthma, liver disorders, and cancer [22]. For instance, the relatively low incidence of coronary heart disease among the French, despite a diet rich in saturated fats, is attributed to the consumption of red wine, which is rich in PCs. These studies attracted considerable public attention, increased consumers awareness of the importance of a “healthy diet” in everyday life, and food industries started to continuously develop new products, defined as “functional food” due to the presence of specific PCs. This inspired scientists to intensively study PCs from plants in terms of their chemical characterization and the evaluation of their biological activities. Thousands of scientific papers related to PCs have been published up to now (according to [22], more than 120,000 articles from 2000 to 2016), and there are several databases providing detailed information on the PC content in different foods (e.g., “Phenol-Explorer” version 1.5.7, INRA and Wishart Research Group, 2009). The most popular journals for covering original research on PCs are The Journal of Agriculture and Food Chemistry, Food Chemistry, PLOS ONE, and Planta Medica. There is an enormous amount of data regarding the biological activities of naturally occurring PCs, among which the most important are antioxidant [23], antimicrobial [24], anticancer [25], anti-inflammatory [26], and neuroprotective activity [27]. Additionally, PCs could be involved in the regulation of different processes in an organism, such as: expression of cell cycle regulatory proteins, signal transduction, or enzyme activity [28]. In addition to being highly bioactive, they express low toxicity, which makes their medicinal use very attractive. 

PCs can affect enzymatic and signalling systems involved in the inflammatory processes, and thus express anti-inflammatory activity. For example, genistein was proven to inhibit tyrosine protein kinase, thus indirectly preventing T-cell proliferation and activation of B cells [22]. Additionally, luteolin, kaempferol, apigenin, and quercetin are powerful inhibitors of β-glucuronidase and lysosomal enzymes released from neutrophils. At the same time, these PCs inhibit phospholipase A2 and prevent the release of arachidonic acid from cell membranes, which is a precursor of proinflammatory mediators [22]. PCs also inhibit enzymes involved in the metabolism of arachidonic acid, such as cyclooxygenases (COX) and lipoxygenases (LOX), and consequently inhibit the production of prostaglandins and leukotrienes, which are essential for the development of inflammatory diseases [23,29,30]. 

One of the cardioprotective mechanisms of PCs is their suppression of platelet activity. Numerous studies have confirmed that PCs can inhibit platelet adhesion, activation, aggregation, and degranulation by acting on different thrombogenic pathways, such as glycoprotein VI (GPVI)–collagen, COX-1–thromboxane, P2Y1/P2Y12–ADP, and protease-activated receptor 1 (PAR1)–thrombin pathways [31].

It has been reported in numerous studies that certain PCs, such as flavan-3-ols, flavanols, and tannins, possess significant antimicrobial (antiviral, antifungal, and antibacterial) activity against many human pathogens, or express synergistic effects with various antibiotics [8]. Additionally, it is known that phenolic acids such as *p*-hydroxybenzoic acid, syringic acid, and gallic, ferulic, and caffeic acid possess high antibacterial activity against several different bacteria [31]. However, the structure–activity relationships and mechanisms underlying the antibacterial activity of PCs, as well as their antibiofilm and antiquorum-sensing activity, have been poorly investigated [32,33].

PCs induce apoptosis and cell cycle arrest and express antiproliferative effects against many human cancer cell lines [9,34]. Thus, certain PCs could be considered as safe and effective agents in cancer prevention and therapy. Additionally, PCs improve the efficacy of chemotherapeutic therapy, reduce the side-effects of chemotherapy, and help to by-pass cancer drug resistance, as well as express antiangiogenic and antimetastatic effects [35,36]. Several studies have confirmed that their anti-cancer efficacy can be enhanced by combining several different PCs instead of using a single one, since they have multiple targets and can exhibit synergistic effects [37]. 

However, most of the data confirming the biological activity of PCs have been obtained from in vitro studies which were performed using pure phenolic compounds (“parent” PCs) or plant extracts rich in PCs. In these studies, the bioavailability and biotransformation of PCs after ingestion were not taken into consideration. It is known that the bioavailability of many PCs after oral intake is very low and that they are extensively metabolized. Thus, it is difficult to obtain reliable conclusions for any “real” health-promoting properties of PCs based on in vitro studies that were carried out with “parent” PCs compounds. Both aspects—biological activity and bioavailability—are rarely investigated at the same time, meaning that the biological activities of PC metabolites, which are actually present in human blood and tissues, are almost fully unknown. Claiming a high biological activity of PCs based on in vitro studies of parent PCs can mislead companies from the food and pharmaceutical industries to develop products rich in PCs that will not exhibit the desired health benefits in humans after oral consumption. Thus, further studies confirming the biological activities of PC metabolites need to be carried out.

### PCs as Antioxidants in Human Health and Disease

Due to their high antioxidant activity, PCs have for a long time been considered powerful agents for protection from oxidative stress and associated pathologies such as cancers, inflammation, cardiovascular, and neurodegenerative diseases. Free radical species occur in the course of numerous physiological processes or under different exogenous factors and can initiate the damage of nucleic acids, lipids, and proteins, resulting in the disturbance of vital cellular functions and causing a wide range of disorders. PCs can exert antioxidant activity by directly scavenging a wide range of highly toxic reactive oxygen species (ROS), suppressing ROS formation by inhibiting enzymes or by complexing metal ions involved in ROS production, and upregulating or protecting the cellular antioxidant defence system [10]. The influence of PCs on the cellular antioxidant defence system is based on the inhibition of xanthine oxidase, the induction of glutathione peroxidase (GPX), catalase, and superoxide dismutase (SOD), and the elevation of endogenous antioxidants [38,39]. By inhibiting lipid peroxidation, PCs protect cell membranes from degradation and enhance the stability of cells against lysis [40]. Additionally, PCs may inhibit the oxidation of low-density lipoprotein (LDL), thus preventing the occurrence of arthrosclerosis [41]. 

Due to their strong antioxidant activity, PCs have many healthful actions in the human body, such as lowering blood pressure and the risk of cardiovascular diseases, and decreasing the occurrence of neurodegeneration and carcinogenesis [28].

The glucuronidation of PCs is considered to be the dominant type of metabolism in the small intestine; thus, glucuronides are the major circulating forms of PCs. However, β-glucuronidases, which cleave glucuronides back to aglycones, have been confirmed in different tissues and body fluids, such as the lungs, liver, and serum, as well as in macrophages and other blood cells [42,43]. Therefore, it is likely that active parent aglycones are present in vivo in a variety of different tissues and cell populations via the action of β-glucuronidases, which reconvert metabolites [44,45].

Additionally, recirculation via enterohepatic pathways (hepatic excretion, enteric deconjugation, and intestinal reabsorption) can be responsible for the slower excretion and longer presence of active PCs in the body [46].

## 3. Bioavailability of PCs

The health properties of PCs are highly dependent on their metabolic transformation in the human body. Since the first data indicating that the bioavailability of PCs can be very low, the interest in evaluating the bioavailability of PCs has increased. For many PCs, their metabolites are identified and quantified in in vitro and in vivo studies [47]. It has been concluded that the chemical structure of PCs dictates their solubility, absorption in the gut, metabolic transformation, and bioavailability. It is, therefore, essential to know the exact chemical structure of the PCs and the amounts in which they are consumed in order to predict their bioavailability. Bioavailability refers to the rate and extent to which a polyphenol is absorbed and becomes available at the site of action. In general, PCs have low bioavailability, meaning that low amounts of unmetabolized polyphenol—the parent molecule—enters the systemic blood circulation and reaches the target tissue. The bioavailability of PCs can vary drastically among different PCs classes, as well as between individual compounds in a particular class.

It is considered that less than 5% of the total PC intake is absorbed and reaches the plasma unchanged [48]. In most cases, these unchanged PCs are undetectable even using highly-sensitive analytical techniques, since their plasma levels are too low to supply target cells with an efficient concentration of PCs [49]. Instead of this, PCs undergo extensive metabolic changes in the intestine or colon, forming a wide variety of new chemical structures (metabolites) [50,51]. After this, the metabolites are absorbed and further metabolized within enterocytes and in the liver by phase I and phase II enzymes (Figure 3). Thus, after ingestion, the PCs metabolites, whose structures can be very different from their parent molecules, reach the target tissue and may or may not be bioactive. PCs are mainly excreted through bile, while renal excretion is usually a minor route of elimination [52].

Following their ingestion, most polyphenol aglycones are absorbed in the small intestine unmetabolized, via passive diffusion from the intestinal lumen into the enterocytes, as they are non-polar molecules (Figure 3). However, PCs are predominantly present in plants in the form of glycosides. Glycosylation patterns determine the digestion, absorption, and metabolism of PC glycosides. Most of them are partially hydrolysed via the enzymatic activity of lactase phloridizin hydrolase (LPH), which is part of the apical brush-border epithelial cells (enterocytes) present in the small intestine. This is then followed by the entrance of the released aglycones into the epithelial cells via the process of passive diffusion. Alternatively, glucosides use sodium-dependent glucose transporter SGLT1 to get imported into enterocytes, where cytosolic β-glucosidase (CBG) hydrolyses them to aglycones. In epithelial cells, the aglycones are partially transformed into sulphate, glucuronide, or methyl conjugates. These aglycone metabolites are then transported to the hepatic portal vein by ATP-binding cassette (ABC) transporters and carried by the blood into the liver, where the conjugation processes are continued. The conjugation of PCs is a metabolic detoxification process that facilitates their biliary and urinary elimination by increasing their hydrophilic character. The resulting conjugates are then transported, together with other intestinal metabolites, into the systemic circulation, from where they will be distributed to the different organs and tissues. The PCs that are not absorbed in the small intestine reach the colon, where the colonic microflora hydrolyses glycosides into aglycones and degrades them into more simple degradation products—for example, 3-hydroxyphenylacetic, 3,4-dihydroxybenzoic, 3,4-dihydroxyphenylacetic, 3-(3-hydroxyphenyl)-propionic, 3-phenylpropionic, and 3-(4-hydroxyphenyl)-propionic acids [10,47,53]. These listed metabolites are further excreted out of the body via the faeces or taken into the blood circulation, where after conjugation in the liver, they would ready again to reach target tissues and express their biological activity. 

In in vivo studies, 23 metabolites of quercetin have been identified. The main metabolites of quercetin in the plasma are quercetin-3′-sulfate, quercetin-3-sulfate, and quercetin-3-glucuronide. However, the urinary metabolites of quercetin have been found to be quercetin-3′-glucuronide, quercetin diglucuronide, isorhamnetin-glucuronide sulphate, isorhamnetin-glucuronide, and isorhamnetin-methyl quercetin diglucuronide [54]. Quercetin-3-*O*-glucoside is absorbed mainly in its native form—the same as rutin (also known as quercetin-3-*O*-rutinoside), which cannot be hydrolysed or glucuronidated in the small intestine at all [55]. The half-lives of elimination of quercetin and its glycosides from the body are considered to be very slow, ranging from 15 to 28 h—indicating that these compounds could be accumulated in tissues long enough to express biological activity [56].

After ingestion of food rich in kaempferol, four different metabolites can be identified, where kaempferol mono- and di-sulphates have been detected only in urine, while kaempferol aglycone and kaempferol-3-glucuronide were found in plasma [10]. 

Apigenin-glycosides are hydrolysed by β-glucosidases in the stomach and small intestines to generate apigenin. Apigenin can be directly absorbed into the systemic blood circulation, or may firstly undergo downstream phase I and II metabolism in the small intestines and liver to generate hydroxylated metabolites such as luteolin, or glucuronidated and sulfonated metabolites [14]. Luteolin 7-*O*-β-glucoside is first hydrolysed to luteolin in the small intestine and then converted to glucuronides by passing through intestinal mucosa, and are absorbed in the form glucuronides, while a certain amount of luteolin is absorbed in the form of free aglycone [57].

After oral intake of naringenin-7-*O*-glucoside, it is hydrolysed by an intestinal enzyme to aglycone naringenin and then glucuronidated within the epithelium, and afterwards, is largely esterified to sulphate in the liver [15,58]. Naringenin-7-rhamnoglucoside (naringin) cannot be hydrolysed by β-glucosidase in the small intestine, but rather only by colonic microflora. Liberated aglycone is then absorbed in the colon [15].

Concerning the bioavailability of flavan-3-ols, it has been observed that after consumption of red wine containing catechins and epicatechins, the concentration of 3′-*O*-methyl-catechin, sulphate, and glucuronide metabolites in plasma increases [59]. It has been shown that epicatechin and catechin are extensively *O*-methylated during their transfer across the jejunum, due to the activity of catechol-*O*-methyltransferases [45]. After absorption by epithelial cells, they may be further glucuronidated, so that they enter the blood circulation in *O*-methylated and glucuronidated forms. Procyanidins (oligomers and polymers of catechin and epicatechin) are unstable under the acidic environment conditions of the gastric milieu and essentially decompose to epicatechin or catechin monomers and dimmers, which are further metabolized by the previously mentioned pathway [60].

Stilbene resveratrol is absorbed after oral consumption to a very high extent (75%) [61]. After absorption, resveratrol is conjugated with glucuronic acid, forming mainly resveratrol-4′-*O*-glucuronide or resveratrol-3-*O*-glucuronide, followed by the formation of resveratrol-4′-*O*-sulfate, resveratrol-3-*O*-sulfate, and resveratrol disulfates in the liver [21,44]. Intestinal bacteria also contribute to resveratrol metabolism, converting it to dihydroresveratrol (which is partially absorbed and conjugated) and two other microbial-derived metabolites—3,4′-dihydroxy-trans-stilbene and 3,4′-dihydroxybibenzyl. Resveratrol is mainly excreted in the urine and faeces [21].

After oral administration, curcumin undergoes limited absorption in the small intestine, followed by metabolism in enterocytes and hepatocytes to dihydrocurcumin, tetrahydrocurcumin (THC—the major metabolite), hexahydrocurcumin (HHC), and octahydrocurcumin (OHC) by alcohol dehydrogenases. Curcumin and its reductive metabolites can be easily conjugated by phase II metabolism to monoglucuronides, monosulphates, and mixed glucuronide/sulphates. Curcumin is excreted through faeces, with minimal elimination in the urine [21].

PCs are commonly mixed with different macromolecules such as carbohydrates, lipids, and proteins, forming a food matrix. There is evidence indicating that food microstructures affect the bio-accessibility and bioavailability of PCs. Thus, urgent research is needed to determine PCs’ bioavailability from different foods [62].

Despite their poor bioavailability, PCs are undoubtedly responsible for many biological effects, which have been proven in vivo—mainly in animals. This low bioavailability/high bioactivity paradox can be explained by the high activity of PC metabolites. The biological activities of PC metabolites have rarely been investigated. As recent studies have shown, metabolites derived from certain dietary PCs elicit significant intrinsic bioactivities that could explain the effects observed for their parent compounds [63,64]. Additionally, the antioxidant and anti-inflammatory activities of some quercetin metabolites (quercetin-3-*O*-glucuronide, 4′-*O*-methylquercetin, and 3′-*O*-methylquercetin (isorhamnetin)) have been investigated. It was shown that all the investigated metabolites demonstrated a notable antioxidant activity, but one lower than that of quercetin aglycone, while 4′-*O*-methylquercetin expressed a significantly superior anti-inflammatory potential compared with quercetin and other metabolites, via its inhibition of the COX and 12-LOX enzymes [65].

The protective effects of epicatechin and one of its major metabolites—3′-*O*-methyl epicatechin—against cell death induced by H_2_O_2_ has been evaluated [60]. The results indicated that both compounds similarly protected cells from death via suppression of caspase-3 activity. Resveratrol metabolites were found to possess in vitro cytotoxic, anti-inflammatory, antioxidant, and delipidating properties [21]. The reductive metabolites of curcumin THC, HHC, and OHC express stronger antioxidant activity than curcumin. Curcumin metabolites also possess anti-inflammatory activity, but weaker than that of curcumin. Furthermore, THC exhibits antiproliferative, antiangiogenic, and chemo-preventive effects, as well as HHC anticarcinogenic effects, while curcumin glucuronide and curcumin sulphate show lower antitumor activity than curcumin. Additionally, THC possesses antidiabetic activity, while HHC contributes to curcumin’s cardioprotective effects, as it possesses anti-platelet aggregation properties and can enhance its anti-atherosclerotic effects [21].

To establish a conclusion on the efficiency of PCs in the prevention of human disease and improvement of human well-being, data regarding its bioavailability and the biological activity of its metabolites are needed. However, in the literature, these data can be found only for very small number of polyphenolic compounds.

## 4. Main Features of Ferroptosis and Its Mechanisms

Cell death is an inevitable part of cell life that can occur under physiological or pathological conditions. Conventionally, it was believed that cell death can occur mainly by two different ways—apoptosis or necrosis [66].

Apoptosis is the best-defined form of controlled” physiological” cell death. Via apoptosis, unwanted cells are eliminated, thus securing normal development, homeostasis, and disease prevention. It is morphologically characterized by cell shrinkage, membrane blebbing, nuclear condensation, and DNA fragmentation. The main biochemical markers of apoptosis are the activation of enzymes caspases and the exposure of phosphatidylserines on the plasma membrane. Caspases break down hundreds of cellular proteins, leading to the loss of cellular functions and cell death, while phosphatidylserines on the plasma membrane invite phagocytes to remove apoptotic cells without inducing an inflammatory response or damage to surrounding tissues [66,67,68].

Necrosis is defined mainly as uncontrolled ”pathological” cell death, characterized by the lysis of acutely injured cells as a response to cellular, chemical, or physical stress. Stress stimuli that can induce necrosis include infection (cytokines, pathogens), hypoxia, oxidative stress (H_2_O_2_), trauma (irradiation), toxins (different drugs), etc. In contrast to apoptosis, necrosis activates an inflammatory response in surrounding tissues due to the spillage of intracellular content [67,69].

New types of cell deaths and their regulation is a hot research topic nowadays. Consequently, a continuously growing list of new and diverse ways by which cell death can happen, be regulated, and even be reversed, has been compiled. Newly introduced ways of regulated cell death can fall into new groups or into subgroups of apoptosis or necrosis [67,70].

One newly introduced process of cell death, distinct from apoptosis or necrosis, is ferroptosis. Ferroptosis was firstly proposed by Dixon et al. in 2012 as a cell death induced by iron and characterised by excessive lipid damage [71]. Explained in more detail, ferroptosis is an iron-dependent, regulated mode of oxidative cell death, characterized by increased lipid peroxidation and the accumulation of toxic lipid peroxides (LOOH), which are considered to be toxic ROS. Accordingly, cell death solely induced by ferroptosis can be reversed by iron chelators, lipophilic antioxidants, and inhibitors of lipid peroxidation. In addition, it has been revealed that ferroptosis has an important role in the development of many chronic diseases, and thus has come to attention as a promising target for new treatments aiming to hinder the progress of related diseases.

### 4.1. Ferroptosis Characteristics and Its Mechanisms of Induction

The morphological characteristics of ferroptosis are reduced mitochondrial volume, increased mitochondrial bilayer membrane density, and decreases in or loss of mitochondrial cristae, while the cell and nucleus membranes remain undamaged. The biochemical markers of ferroptosis are decreases in the concentration of intracellular glutathione (GSH) and reductions in the activity of GPX4. As a consequence—since LOOH cannot be metabolized by the GPX4-catalyzed reduction reaction—lipid peroxidation progresses, resulting in increased amounts of intracellular lethal lipid–ROS, which consequently leads to ferroptosis. It is important to emphasize that GPX4, among all other GPXs in the cell, is the only GPX able to directly reduce LOOH within biological membranes and therefore plays a crucial role in regulating the redox state of the cell membrane, level of lipid peroxidation in membrane, and, consequently, ferroptosis [72]. From a genetic point of view, ferroptosis is a process regulated by multiple genes that are mainly of importance to iron and lipid peroxidation metabolism, but its exact regulatory mechanisms are still to be resolved. The role of iron in the development of ferroptosis is still unclear. Namely, ferroptosis cannot be described solely by increases in ROS production derived from the iron-dependent Fenton reaction. Ferroptosis is also connected with iron-dependent enzymes which are part of a cell oxidative process fatal to cells [71,73,74,75].

There are a couple of mechanisms by which ferroptosis can be induced. However, the main method of ferroptosis induction is the inhibition of transportation system Xc- and GPX4 activity [76]. Mechanisms of ferroptosis induction and inhibition are presented in Figure 4.

System Xc- is an amino acid antiporter system present in the cellular phospholipid bilayer which facilitates the entry of extracellular cystine into cells and the exit of intracellular glutamate out of the cell at a ratio of 1:1 [77]. Cystine is intracellularly reduced to cysteine, which later takes part in GSH synthesis. GPX4 is part of the cell’s antioxidative enzyme portfolio and reduces toxic LOOH to their non-toxic corresponding alcohols, thus protecting the cell from oxidative damage. GSH is an essential coenzyme for GPX4 activity, and during the GPX4 catalysed reaction, GSH is converted to its oxidized disulphide form [78].

The major limiting step in GSH synthesis is the availability of cysteine. Thus, the inhibition of system Xc- activity reduces the synthesis of GSH, which leads to a decrease in GPX4 activity and the accumulation of LOOH, and, unfortunately, to the occurrence of oxidative damage and ferroptosis [74,78]. System Xc- can be inhibited, and thus ferroptosis induced, by a couple of compounds such as erastin—a new small molecule by whose introduction ferroptosis was firstly discovered, and its analogues imidazole ketone erastin and piperazine erastin [71,75,79], tumour protein P53 [80,81], and sulfasalazine— a drug used regularly in the treatment of rheumatoid arthritis, ulcerative colitis, and Crohn’s disease, and a promising anti-cancer target [82,83].

As written above, the inhibition of GPX4 activity leads to a rise in cytosolic LOOH—a primary marker of ferroptosis. It has been confirmed that upregulation of GPX4 expression hinders ferroptosis, while downregulation of GPX4 expression makes cells more prone to ferroptosis. The same is confirmed with compounds RSL3, DPI7, and DPI10, which directly inhibit GPX4 activity and thus induce ferroptosis. In addition, it is known that the obstruction of the mevalonate pathway blocks the synthesis of selenocysteine tRNA, an essential amino acid present in GPX4 structure, and so impairs GPX4 activity and induces ferroptosis [73,84,85,86].

Furthermore, there are additional mechanisms that are proven to have an effect on ferroptosis progression, and knowledge in this field is gradually emerging.

Namely, voltage-dependent anion channel (VDAC) channels have a main role in the passive diffusion of anionic hydrophilic mitochondrial metabolites across the outer membrane [87]. It has been confirmed that VDAC opening, induced by erastin, increases mitochondrial ROS and lipid peroxidation and alters the permeability of the outer mitochondrial membrane, consequently leading to cell death by ferroptosis [84].

The p53 tumour suppressor is ‘the guardian of the genome’, which takes part in the control of cell reactions to different traumas, such as malnourishment, DNA injury, hypoxia, and oncogene stimulation. p53 initiation can lead to cell survival or programmed cell death, determined by the type and level of trauma. Unfortunately, p53 is usually mutated or depleted in many cancers. p53 also regulates ferroptosis either through a transcriptional or post-translational mechanism. On one hand, p53 can enhance ferroptosis, but on the other hand, p53 suppresses ferroptosis depending on the situation [81,85,86].

Coenzyme Q10 (CoQ10) is a lipophilic antioxidant that the body produces naturally. CoQ10 has dual role—it is essential for electron transfer in the electron respiratory chain in the mitochondrial membrane, and it is free radical-scavenging antioxidant in the plasma membrane. In order for CoQ10 to express its roles, it is necessary that CoQ10 undergoes a redox cycle from fully oxidized ubiquinone to be effectively, fully reduced to ubiquinol. Recently, a new oxidoreductase—FSP1—has been revealed, which reduces CoQ10 at the plasma membrane. Thus, depletion of CoQ10 or inhibition of FSP1/CoQ10 would lead to ferroptosis occurrence, and vice versa. Furthermore, the FSP1/CoQ10 system is able to suppress lipid peroxidation alone and acts in parallel to GPX4 to inhibit ferroptosis [87,88]. In addition, it was recently confirmed that supplementation with dihydroorotate—which is a substrate of dihydroorotate dehydrogenase (DHODH), the enzyme which reduces ubiquinone to ubiquinol—inhibits ferroptosis independently of the GSH/GPx4 and FSP1/CoQ10 systems. This suggests that the activation or maintenance of DHODH activity is an additional ferroptosis defence mechanism in the mitochondria [89].

### 4.2. Links between Lipid Peroxidation, ROS, and Ferroptosis

As mentioned before, the most important features of ferroptosis are excessive lipid peroxidation and raised levels of LOOH in the cell. Lipid peroxidation is by definition a free radical chain reaction under which oxidants, such as free radicals, attack lipids containing carbon–carbon double bond(s)—particularly polyunsaturated fatty acids (PUFAs)—and thus fragment their structure, mainly in cell membranes, resulting in cell damage [76,90,91].

Lipid peroxidation has three phases: initiation, propagation, and termination. Initiation of lipid peroxidation starts with an electron transfer from a reduced transition metal, such as Fe^2+^, to a LOOH in a Fenton-like reaction, producing the alkoxy radical (LO^•^; Equation (1)). Afterwards, LO^•^ either extracts an H from a new lipid molecule (L-H/PUFA), creating a carbon-centred alkyl radical (L^•^), or rearranges, forming an epoxy group and a new carbon-centred radical in the same fatty acid chain (Epoxy-L^•^; Equation (2)). LO^•^ typically affects PUFAs, because they contain multiple double bonds in between which lie methylene groups (-CH2-) that possess highly reactive H atoms. Oxygen is than added to radicals, L^•^ or Epoxy-L^•^, forming a lipid hydroperoxyl radical (LOO^•^ or Epoxy-LOO^•^; Equation (3)). From LOO^•^, LOOH is generated by an H transfer, for instance from L-H/PUFA, when a new radical is formed L^•^, which is then susceptible to O_2_ addition—thus further propagating lipid peroxidation (Equation (4)). In the presented equations, ROS—in the form of lipid radicals or peroxides—are continuously produced, making them able to degrade lipid structures in the cell—mainly in cell membranes—resulting in cell damage and ferroptosis. Bearing in mind all these reactions, the direct link between ROS and ferroptosis is more than obvious [90,92,93].

LOOH + Fe^2+^ → LO^•^ + Fe^3+^ + H_2_O (1)

LO^•^ + L-H → L-OH + L^•^ (Epoxy-L^•^) (2)

(Epoxy-L^•^) L^•^ + O_2_ ←→ LOO^•^ (Epoxy-LOO^•^) (3)

LOO^•^ + L-H → LOOH + L^•^ (4)

LOO^•^ + Aox-OH → LOOH + Aox-O^•^ (5)

There are a couple of scenarios by which the lipid peroxidation can be terminated. Lipid radicals such as LOO^•^ could be neutralized by lipophilic antioxidants (H donors); for example, by coenzymes Q or tocopherols (Equation (5)). Additionally, radicals could be neutralized by radical–radical interactions. However, for lipid peroxidation to stop, the presence of free Fe^2+^ must also at a minimum, since Fe^2+^ would propagate the formation of lipid radicals over and over again—as in Equation (1) [91,92,94].

LOOH are unavoidably produced in the cell as a result of oxygen activation during aerobic metabolism. It is not strictly defined how LOOH are produced in the cell, but it is assumed that the first L^•^ in the cell membrane develops the first LOOH after O_2_ addition—which then initiates lipid peroxidation inside the membrane—and is produced via H abstraction from PUFAs by O_2_^•−^. The major biological sources of O_2_^•−^ are NADPH oxidases and mitochondria, where leakage of electrons from the transport chain occurs regularly. Thus, it is no surprise that mitochondria are the site where the formation of LOOH from which lipid peroxidation is initiated occurs—particularly when reduction by GPX4 is limiting. Additionally, the morphological characteristics of ferroptosis mainly refer to changes in mitochondrial structure, which seems logical if we know that lipid peroxidation is more likely to occur and propagate in mitochondria membranes [76,90,91].

However, at the same time, LOOH are continuously reduced by GPX4 in the presence of GSH. As such, it is until the moment when the GPX4 reaction becomes limiting that Fe^2+^ initiates lipid peroxidation, leading to cell death by ferroptosis. Any mechanism which lowers ROS, LOOH, and LOO^•^ production and the accumulation of iron will result in the hindering of ferroptosis. On the other hand, it could be implied that ferroptosis is the result of the malfunction of cell antioxidant mechanisms controlling oxygen metabolism and toxicity. These mechanisms must be continuously functional and keep reactions propagating lipid peroxidation at low rates. Otherwise, ferroptosis is likely to occur.

Lately, it is believed that enzymes LOXs are also enhancers of ferroptosis. Namely, LOXs are dioxygenases that catalyse the formation of LOOH from PUFAs such as linoleic acid and arachidonic acid. LOXs are involved in the production of a range of different LOOH which, in the end, lead to the formation of inflammatory lipid mediators involved in the development of many diseases [92,93]. LOOH produced by LOXs could also be involved in the development of lipid peroxidation and ferroptosis. Furthermore, it is known that antioxidants, such as tocopherols/Vitamin E, inhibit LOX activity by scavenging free radical intermediates at the LOX catalytic site. Thus, the question has been asked whether ferroptosis inhibition by antioxidants could be due to LOX inhibition. However, the link between LOX and ferroptosis, as well as the application of LOX inhibitors as therapeutics in the hindering of ferroptosis-related diseases, is still to be defined [90,94].

## 5. Ferroptosis in Diseases

The past decade has brought us immense knowledge on ferroptosis and its association with many chronic diseases—mainly cardiovascular diseases, neurodegenerative diseases, and cancer. These findings have initiated the development of new therapeutical strategies for the treatment of these conditions, where inhibition of ferroptosis in treating cardiovascular and neurodegenerative diseases is considered a prime goal. On the other hand, the selective induction of ferroptosis has been considered as a potential therapeutical strategy in some types of cancers [86,93].

Inhibitors of ferroptosis could be classified into one of several listed groups: lipophilic free radical-trapping antioxidants (ferrostatins, liproxstatins, α-tocopherol, butylated hydroxytoluene (BHT), N-acetylcystein), iron chelators (deferoxamine mesylate, deferoxamine, deferiprone), deuterated polyunsaturated fatty acid phospholipids (PUFA-PLs), and dihydroorotate—a substrate of DHODH [89,93,95]. These have been proven to prevent ferroptosis by increasing GSH levels, activating GPX4, directly inhibiting lipid peroxidation, or by reducing ubiquinone to ubiquinol—a powerful antioxidant form of CoQ10—in the mitochondrial membrane [89]. The efficacy of these inhibitors in hindering ferroptosis and preventing diseases has been confirmed mainly in cell lines. Whether compounds targeting the induction or inhibition of ferroptosis have high specificity and minimal side effects remains to be elucidated in preclinical and clinical studies to come.

### 5.1. Cardiovascular Diseases and Ferroptosis

It is well known that increases in LOOH and ROS, as well as lipid peroxidation propagation, lead to chronic inflammation in macrophages, vascular smooth muscle cells, and vascular endothelial cells, and the formation of foam cells—which eventually leads to the formation of atherosclerotic lesions [96,97]. In addition, it has been confirmed that increased iron amounts support the development of atherosclerosis by inducing lipid peroxidation both in vitro and in vivo [98,99]. Additionally, increased LOOH caused by ox-LDL accumulation within atherosclerotic plaques is a fruitful setting for the initiation of ferroptosis [100]. Before ferroptosis was discovered, it was known that overexpression of GPX4 significantly slows down the progression of atherosclerotic plaques in mice [101]. All these results directly connect the initiation of atherosclerosis with ferroptosis, which is nowadays considered a fact [96,102]. Consequently, it has been proven in an in vivo animal model that the inhibition of ferroptosis by antioxidant ferrostatin-1 constrains atherosclerosis via the reduction of lipid peroxidation [97].

Similar findings—regarding increased levels of LOOH, ROS, and lipid peroxidation, as well as reductions in GSH levels and GPX4 activity—were observed in the development of stroke and ischemia/reperfusion injury. Obviously, ferroptosis contributes to the development of different cardiovascular diseases with similar mechanisms. 

All these findings contribute to a deeper understanding regarding the biochemical mechanisms of cardiovascular disease development and suggest ferroptosis as a new therapeutic target for treating them [93,96].

### 5.2. Neurodegeneration and Ferroptosis

Before the concept of ferroptosis was introduced, neurodegenerative diseases were believed to be triggered by apoptosis [103]. Since 2012, when ferroptosis as new sort of cell death was defined, there has been a great number of studies supporting the belief that ferroptosis is undoubtedly linked to the development of neurodegenerative diseases. Namely, it has been confirmed in numerous studies that neurodegenerative diseases, such as Alzheimer’s disease, Parkinson’s disease, Huntington’s disease, or Amyotrophic lateral sclerosis, are followed by iron accumulation and elevated oxidative stress—particularly lipid peroxidation—in specific regions of the central and/or peripheral nervous system, which are all known markers of ferroptosis. In addition, many neurological diseases are characterized by a decrease in GSH and GPX4 levels, which is followed by an immense creation of ROS in neuronal cells, finally leading to oxidative injury of different biomolecules and death by ferroptosis [86,93]. On the other hand, it has been shown that inhibition of ferroptosis by antioxidants (e.g., vitamin E) or iron chelators (e.g., deferoxamine, deferiprone) in neurons can effectively slow down the progress of these listed neurodegenerative diseases, confirming the clear link between ferroptosis and the development of neurodegeneration.

Clinical trials have already shown that Alzheimer’s disease patients who receive vitamin E in doses of 2000 IU/day show a slower decline in cognitive function compared to a placebo group [104]. Additionally, a great body of preclinical evidence shows that intranasal treatment with deferoxamine improves memory and behavioural outcomes in rodent models of Alzheimer’s and Parkinson’s diseases [105]. Additionally, clinical studies of deferiprone have shown that symptoms of early Parkinson’s diseases could be decreased by the reduction of iron levels in patients [106]. At the moment, deferoxamine and deferiprone are approved and used drugs in iron accumulation disorders. They are also strong drug candidates for managing neurodegeneration, but their dosing regimens must be standardized in order for them to be routinely applied as therapeutic agents [105,107]. Moreover, PCs, which are also strong antioxidants and iron chelators, have been proven to decrease the development of neurodegeneration. Epigallocatechin gallate, a polyphenol from green tea with iron chelating and antioxidant abilities, have been proven to delay the development of Parkinson’s disease in animal models [108].

The therapeutic value of the application of inhibitors of ferroptosis in the treatment of neurodegenerative diseases is still to be defined, but it is strongly believed that it will lead to advancements in the management of neurodegeneration.

### 5.3. Cancer and Ferroptosis

At the moment, it is believed that ferroptosis has a dual role in tumour development, since it can promote tumour promotion and suppression. What effects on tumour development ferroptosis would have depends on the type and amount of released damaged molecules and the activation of the immune response triggered by ferroptosis damage. Even though it is known that most cancer cells exhibit increased levels of ROS, and that they are dependent on the presence of glutathione in order to maintain their survival and proliferation—which could be linked to ferroptosis—the exact mechanisms of ferroptosis and cancer cross-talk is still to be discovered [109,110].

Today, the most investigated link between ferroptosis and cancer is concentrated on the selective implications of ferroptosis in the hindering of cancer development. Namely, the treatment of cancer is mainly based on inducing cancer cell death. Since ferroptosis is a novel method of cell death, its selective induction in cancer cell lines is considered a strategy for new therapies. Even though most of the research regarding this strategy is performed in vitro, results are showing great potential for the future. Up to now, the induction of ferroptosis has mainly been shown by depleting GSH and CoQ10, the inactivation of GPX4, by increasing iron availability, and by promoting oxidative stress and lipid peroxidation [86,111,112]. By different compounds and their combinations, such as artesunate, cotylenin A, phenylethyl isothiocyanate, piperlongumine, erastin, cisplatin, sulfasalazine, sorafenib, and low-density lipoprotein–docosahexaenoic acid, the proliferation of different types of cancers could be inhibited. These listed compounds have been shown to be promising in treating hepatocellular carcinoma [113,114], ovarian cancer [115], colorectal cancer [116], lung cancer [117], gastric cancer [118], and pancreatic cancer [119,120]. At the moment, some of the listed compounds which induce ferroptosis in cancer cells are already FDA-approved for the treatment of different diseases, such as sorafenib (treatment of kidney, liver, and thyroid cancer), sulfasalazine (anti-rheumatic drug), artesunate (antimalarial agent), and lanperisone (muscle relaxant) [93]. Additionally, it is strongly believed that ferroptosis could increase the efficacy of chemotherapy, and thus combinations of these two could improve the outcomes of cancer therapy in the future [109].

## 6. Inhibition of Ferroptosis by PCs

PCs have also been identified as ferroptosis potent inhibitors, as confirmed in numerous in vitro and in vivo studies in different disease models. In Table 1, the available data regarding PCs’ potential to inhibit induced ferroptosis in vitro and/or in vivo are given in detail. Listed references were obtained from an in-depth search using the PubMed^®^ literature database, using the keywords (different combinations of listed words): polyphenols, phenols, natural products, ferroptosis, ferroptosis inhibition, anti-ferroptosis, oxidative stress, lipid peroxidation, in vitro, and in vivo. Additionally, after finding each reference relevant to this review option, options Cited by and Similar articles was also used to find additional relevant publications.

Even though some could consider that due to the low bioavailability of PCs and their excessive metabolism in vivo they would not express potent anti-ferroptosis activity in vivo as they did in in vitro system, the studies show opposite. The PCs listed in Table 1 are also listed on Figure 1, regarding the classes of PCs to which they belong.

It should be emphasized that the listed references in Table 1 are only those where in vitro ferroptosis is induced by a known ferroptosis inducer, such as erastin, followed by treatment with corresponding PCs and, afterwards, the detection of ferroptosis biochemical markers. Regarding in vivo studies, the listed references are those that followed the intentional inhibition of ferroptosis by PCs in models where ferroptosis was not induced by a known ferroptosis inducer, but rather with a procedure known to cause tissue failure by increasing oxidative stress, which was then followed by an elevation of all known markers of ferroptosis. However, there is great possibility that there are many in vivo studies which are not listed here, but that present solid evidence that PCs inhibit ferroptosis. Namely, in many in vivo studies, reductions in standard ferroptosis markers were evaluated due to polyphenol treatment after application of different procedures which elevate oxidative stress, but the authors did not explicitly state that ferroptosis was reduced; rather, they stated that oxidative stress or inflammation was inhibited. The reason for this occurrence is that ferroptosis is a new phenomenon characterized by the same biochemical markers as oxidative stress or inflammation, whose markers have been enormously studied over recent decades. To summarize, it should be expected that there is much more in vivo evidence which confirms that PCs inhibit ferroptosis than is listed here, which additionally raises the importance of PCs as potential drug candidates for the many chronic diseases that are induced by ferroptosis.

Interestingly, in couple of the studies listed in Table 1, it was shown that PCs (e.g., quercetin) are selective inhibitors of ferroptosis, since they did not rescue cell viability from induced apoptosis, necrosis, or autophagy [132]. Additionally, it was confirmed that different PCs isolated from the plants *Hypericum japonicum* and *Cullen corylifolium*, or whole plant extracts from *Baccharis dracunculifolia*, have anti-ferroptosis effects, but this effect was only confirmed by cell viability tests after ferroptosis was induced in vitro [121,154,155]. 

From taking a closer look at Table 1, it could be seen that the inhibition of ferroptosis by different PCs is greatly based on the same mechanisms, which are closely related to decreasing oxidative stress—inhibition of ROS production, lipid peroxidation, iron accumulation, GSH depletion, LOX activity, or increased expression of GPX4 and Nrf2 (Figure 5). The mechanisms of PC inhibition of ferroptosis could be divided in three main types of activities: (i) neutralization of ROS and other free radicals by donating an electron or hydrogen atom from free hydroxyl groups; (ii) chelation of Fe; (iii) activation of transcription factors, such as Nrf2.

Most of PCs’ anti-ferroptotic activities are based on PCs’ ability to neutralize ROS and other free radicals. Namely, PCs’ hydroxyl groups are great hydrogen donors, which in reaction with free radicals, lead to the termination of the cycle of generation of new radicals. In this process, the corresponding PC radical form is formed, which is chemically stabilized since free e^-^ at the hydroxyl group is delocalized within the aromatic ring structure. Correspondingly, PC radicals are not an oxidative threat to the functional biomolecules in the cell [38].

Additionally, PCs are powerful chelators of Fe ions, making them unable to further produce reactive species such as ROS or different lipid radicals, which inevitably leads to ferroptosis suppression. Furthermore, even though the exact mechanism of how PCs inhibit LOX activity is not known, it is speculated that LOX inhibition by PCs could be due to the reduction of Fe^3+^ in the LOX activity centre by PCs [65].

In a number of the above listed studies, it was confirmed that PCs regulate the Nrf2/System xc-/GPX4 axis and thus decrease ferroptosis (Figure 5). This came as no surprise, since Nrf2 is a universal transcription factor which binds to the antioxidant response elements (ARE) of different genes and increases their expression. Thus, among others, Nrf2 triggers the synthesis of antioxidant enzymes and cytoprotective genes as a response to oxidative stress. Namely, it is known that some PCs bind to Keap1 and inactivate it. Keap1 has an important role in the ubiquitination and degradation of Nrf2. Thus, after PCs inactivate Keap1, the half-life of Nrf2 is extended and its binding to ARE is more certain and longer-lasting, leading to the reduction of oxidative stress and ferroptosis [156]. Interestingly, Nrf2—via binding to ARE, among other genes—activates the expression of glutathione synthetase, a crucial enzyme required for the synthesis of GSH as well as GPX4 [157].

Additionally, it can be seen that most of these studies were conducted in the last three years, which shows that PCs’ effects on ferroptosis inhibition is a hot topic at the moment.

## 7. Conclusions

At this moment, the relevance of ferroptosis for the development of different human disease is an active research area, and the development of innovative approaches and compounds for identifying novel therapeutics that target the ferroptosis pathway in humans could not be timelier. Plants are exposed to a variety of stresses, such as oxidative stress, that trigger physiological changes similar to those found in ferroptosis. As a result, plants produce a great diversity of biochemicals, including PCs, in order to fight oxidative stress and its physiological consequences. Since they do this successfully, many of those compounds could be relevant to human medicine. Therefore, plants, and thus PCs, represent an excellent source of anti-ferroptotic compounds whose real potential in fighting ferroptotic-related diseases in humans is still to be revealed. In order to be successful, researchers need to perform more research regarding the screening and in vivo confirmation of PCs’ anti-ferroptotic potential, bearing in mind their effectiveness, bioavailability, non-toxic doses, and methods of application, in order to enable their clinical application.

## Figures and Tables

**Table 1:** PCs as potent inhibitors of ferroptosis, proposed mechanisms for ferroptosis inhibition, and model systems where ferroptosis inhibition has been proven.

Polyphenol	Proposed Mechanisms of Ferroptosis Inhibition	Model System in Which Ferroptosis Inhibition Has Been Proven		Refs.
		In Vitro	In Vivo	
Phenolic acids				
Artepillin C	decreased ROS production	mouse hippocampal neuronal cell line (HT-22)	/	[121]
Gastrodin	decreased GSH depletion, lipid peroxidation, iron accumulation, and ROS production, and upregulation of GPX4	rat glioma cell line (C6), where ferroptosis was induced by H_2_O_2_	/	[122]
Flavonoids				
Baicalein	decreased lipid peroxidation, iron accumulation, and GSH depletion, and GPX4 degradation	human pancreatic adenocarcinoma cell lines (PANC1; BxPc3)	*/*	[123]
	12/15-LOX inhibition, decreased lipid peroxidation and ROS production	human T lymphoblastic leukaemia cell lines (Molt-4; Jurkat)	*/*	[124]
	12/15-LOX inhibition, decreased lipid peroxidation and ROS production, and upregulated expression of GPX4	mouse hippocampal neuronal cell line (HT-22)	*/*	[125]
	decreased phosphatidylethanolamine oxidation	*/*	mice were treated with baicalein with intraperitoneal injection after ferroptosis was induced by traumatic brain injury. Ferroptosis was examined in pericontusional cortex tissue	[126]
Baicalin	decreased lipid peroxidation, ROS, and iron accumulation, and increased expression of GPX4	rat cardiac myoblast cells (H9c2)	rats were treated with baicalin orally before myocardial ischemia/reperfusion injury was induced. Ferroptosis markers were evaluated in heart tissue	[127]
Epigallocatechin-3-gallate	decreased iron accumulation, ROS production, lipid peroxidation, and GSH depletion, and increased expression of GPX4	rat cardiac myoblast cells (H9c2)	*/*	[128]
	increased expression of GPX4, maintained normal mitochondria structure, decreased lipid peroxidation, and GSH depletion	*/*	mice were treated with epigallocatechin-3-gallate orally after ferroptosis was induced with doxorubicin. Ferroptosis markers were evaluated in heart tissue	[128]
	decreased iron accumulation, lipid peroxidation, and GSH depletion, and increased expression of GPX4	mouse pancreatic β-cell line (MIN6)	*/*	[129]
Quercetin	decreased GSH depletion, lipid peroxidation, iron accumulation, and ROS production, and upregulated expression of GPX4. Maintained normal mitochondria structure and SOD activity	*/*	mice were treated with quercetin orally after type 2 diabetes was induced. Ferroptosis markers were evaluated in pancreatic tissue	[130]
	decreased ROS production, lipid peroxidation, and GSH depletion	rat insulinoma cell line (INS-1)	*/*	[130]
	decreased lipid peroxidation	bone marrow-derived mesenchymal stem cells	*/*	[131]
	increased GSH levels, decreased lipid peroxidation, and GPX4 depletion	/	mice were treated with quercetin orally before acute kidney injury was induced. Ferroptosis markers were evaluated in kidney tissue	[132]
	maintained normal mitochondria structure, decreased GSH depletion, lipid peroxidation, and ROS production	rat kidney epithelial cell line (NRK-52E) and human proximal tubular cell line (HK-2)	*/*	[132]
Galangin	decreased lipid peroxidation, GSH depletion, and iron accumulation, and increased expression of GPX4	*/*	gerbils were treated with galangin orally after cerebral ischemia-reperfusion injury was induced. Ferroptosis markers were evaluated in hippocampal coronal tissue	[133]
Kaempferide	decreased ROS production and increased ARE activation	mouse hippocampal neuronal cell line (HT-22)	/	[121]
Keampferol	decreased ROS production and increased ARE activation	mouse hippocampal neuronal cell line (HT-22)	*/*	[121]
	decreased iron accumulation, lipid peroxidation, and ROS production, and increased expression of GPX4 and NRF2. Maintained normal mitochondria structure	primary mouse cortical neurons where ferroptosis was induced by oxygen–glucose deprivation/reoxygenation	*/*	[134]
Icariin	decreased lipid peroxidation, iron accumulation, and upregulated expression of GPX4 and Nrf2	human synoviocyte cell line (HUM-CELL-0060)	*/*	[135]
	decreased lipid peroxidation, iron accumulation, and ROS production, and upregulated expression of GPX4 and NRF2	rat cardiac myoblast cells (H9c2) where ferroptosis was induced by hypoxia/reoxygenation	*/*	[136]
Puerarin	reduce lipid peroxidation, iron accumulation, and expression of NOX4. Increased GPX4 expression	rat cardiac myoblast cells (H9c2)	*/*	[137]
	reduced lipid peroxidation and expression of NOX4. Increased GPX4 and ferritin expression	*/*	rats were treated with puearin by subcutaneous injection after heart failure was induced by pressure overload. Ferroptosis markers were evaluated in cardiac tissue	[137]
Cyanidin-3-glucoside	decreased ROS production, lipid peroxidation, and iron accumulation, and increased expression of GPX4	rat cardiac myoblast cells (H9c2) where ferroptosis was induced by oxygen glucose deprivation/re-oxygenation	rats were treated with cyanidin-3-glucoside with intraperitoneal injection before myocardial ischemia-reperfusion injury was induced. Ferroptosis markers were evaluated in heart tissue	[138]
Sterubin	decreased GSH depletion, ROS accumulation, and free iron concentration	mouse hippocampal neuronal cell line (HT-22)	/	[139]
Naringenin	Decreased lipid peroxidation, ROS production, GSH depletion, iron accumulation, and expression of NOX4, and increased expression of GPX4 and Nrf2	*/*	rats were treated with naringenin orally before myocardial ischemia-reperfusion injury was induced. Ferroptosis markers were evaluated in myocardial tissue	[140]
Stilbenes				
Resveratrol	decreased lipid peroxidation and iron accumulation, and increased expression of GPX4	rat cardiac myoblast cells (H9c2)	rats were treated with resveratrol orally before myocardial ischemia/reperfusion injury was induced. Ferroptosis markers were evaluated in heart tissue	[141]
Piceatannol	decreased ROS accumulation	rat bone marrow-derived mesenchymal stem cells bmMSCs	/	[142]
Astringin	decreased ROS accumulation	rat bone marrow-derived mesenchymal stem cells bmMSCs	/	[142]
Lignans				
Nordihydroguaiaretic acid	5-LOX inhibition, decreased lipid peroxidation and ROS production	human T lymphoblastic leukemic cells (Molt-4; Jurkat)	/	[124]
Coumarins				
Fraxetin	decreased GSH depletion, iron accumulation, and lipid peroxidation, and upregulated GPX4 and Nrf2	rat cardiac myoblast cells (H9c2) where ferroptosis was induced by oxygen glucose deprivation/re-oxygenation	rats were treated with fraxetin by intraperitoneal injection before myocardial infarction was induced. Ferroptosis markers were evaluated in heart tissue	[143]
Diarylheptanoids				
Curcumin	decreased iron accumulation, lipid peroxidation, and GSH depletion, and increased expression of GPX4	mouse pancreatic β-cell line (MIN6)	/	[129]
	decreased lipid peroxidation and GSH depletion	*/*	mice were treated with curcumin with intraperitoneal injection after ferroptosis was induced by rhabdomyolysis. Ferroptosis markers were evaluated in kidney tissue	[144]
Antraquinons				
Chrysophanol	decreased iron accumulation and ROS production, and increased expression of GPX4	human renal proximal tubular epithelial cell line (HK-2 cells) where ferroptosis was induced with hypoxia/reoxygenation	/	[145]
Chalcons				
Butein	inhibition of lipid peroxidation	rat bone marrow-derived mesenchymal stem cells	/	[146]
Isoliquiritigenin	decreased lipid peroxidation and ROS production, and increased expression of GPX4 and xCT (subunit of x_c_^–^ transporter). Maintained normal mitochondria structure	human proximal tubular cell line (HK-2)	/	[147]
	decreased lipid peroxidation, ROS production, and iron accumulation and increased expression of GPX4	*/*	mice were treated with isoliquiritigenin orally before acute kidney injury was induced. Ferroptosis markers were evaluated in kidney tissue	[147]
Carthamin	decreased lipid peroxidation, ROS production, GSH depletion, and iron accumulation, and increased expression of GPX4	*/*	rats were treated with carthamin orally before cerebral ischemia-reperfusion injury was induced. Ferroptosis markers were evaluated in brain tissue	[148]
Morachalcone D	decreased GSH depletion, ROS production, and iron accumulation, and upregulate GPX4 and NRf2 mRNA.	mouse hippocampal neuronal cell line (HT-22)	/	[149]
Condensed tannins				
Chebulagic and chebulinic acids	decreased ROS accumulation	rat bone marrow-derived mesenchymal stem cells bmMSCs	/	[150]
Geraniin	decrease ROS accumulation, iron accumulation, and lipid peroxidation	rat bone marrow-derived mesenchymal stem cells bmMSCs	*/*	[151]
Other				
Carnosic acid	decreased GSH depletion, lipid peroxidation, iron accumulation, and ROS production, and upregulated expression of Nrf2	rat adrenal gland irregularly shaped cell line (PC-12)	*/*	[152]
Gossypol	decreased iron accumulation, lipid peroxidation, and ROS production	rat cardiac myoblast cells (H9c2)	/	[153]
	decreased lipid peroxidation and increased expression of GPX4	ferroptosis was induced in rat hearts by ischemia/reperfusion followed by treatment with gossypol acetic acid	*/*	[153]
